# Research on the Contour Modeling Method of Peripheral Nerve Internal Fascicular Groups During the Non-Splitting/Merging Phase and Distribution Rules of Model Parameters

**DOI:** 10.3389/fncel.2022.860103

**Published:** 2022-05-13

**Authors:** Yingchun Zhong, Zhihao Tian, Peng Luo, Siyu Sun, Shuang Zhu

**Affiliations:** ^1^School of Automation, Guangdong University of Technology, Guangzhou, China; ^2^Department of Bone and Joint Surgery, Shenzhen Sixth People's Hospital, Shenzhen, China; ^3^Department of Pathophysiology, School of Basic Medical Sciences, Zhengzhou University, Zhengzhou, China

**Keywords:** peripheral nerve bundles in non-splitting and merging stage, Fourier, contour modeling, Hausdorff distance, peripheral nerve repair

## Abstract

**Objectives:**

To investigate benchmark data for docking the same functional nerve bundles based on the mathematical contour model of peripheral nerve internal fascicular groups.

**Materials and Methods:**

First, the discrete points of the original contours of nerve bundles were extracted into a dataset through the image process. Second, two indicators were employed to evaluate the modeling precision. Third, the dataset was modeled by the 3rd-order quasi-uniform B-spline method. Fourth, the dataset was modeled by the Fourier transform method. Fifth, all contours were modeled by the 4th-order Fourier method. Then, the histogram of each parameter from the Fourier model was calculated. Furthermore, the probability density function was fit to each parameter.

**Results:**

First, the optimized sampling number of the 3rd-order quasi-uniform B-spline method is 21. The sampling number is the control point number of the 3rd-order quasi-uniform B-spline, which produces more than 63 parameters in the model. Second, when the Fourier transform model is employed to model the contour of nerve bundles, the optimized order number yields a 4th-order Fourier model, which has 16 parameters. Third, when all contours are modeled by the 4th-order Fourier model, the statistical analysis shows that (1) the pitch parameters a1 and d1 obey the mixed Gaussian distribution; (2) the harmonic parameter b3 obeys the normal distribution; and (3) the pitch parameters b1 and c1 and the remaining harmonic parameters obey the *t* distribution with position and scale.

**Conclusion:**

This work paves the way for the exploration of the correlation between model parameters and spatial extension.

## Introduction

Peripheral nerve injury in the limbs is a common disease in surgical clinics (Sullivan et al., 2016; Zhu et al., 2018). Connecting nerve bundles according to the original anatomical structure will restore nerve conduction and function to the greatest extent during peripheral nerve repair (Zhong et al., 2015, 2020). The contour information of the nerve bundle can provide a positioning reference for the docking nerve (Zhuo et al., 2016; Zhong et al., 2020). At present, research on the modeling method of neural bundle contours is exceptionally scarce. Therefore, exploring the modeling method of the nerve bundle contour in the peripheral nerve and the rules of the model parameters is of great significance to the repair of peripheral nerve injury.

The contours of the nerve bundles are a series of irregular circular curves with different sizes and shapes in the MicroCT image. Zihao et al. (2016) proposed a contour fitting method based on a piecewise cubic polynomial Bezier curve using the modeling method of quasi-circular contours. The result has high accuracy and a good fitting effect. In addition, Zhong et al. (2021) proposed a tensor B-spline method with an arbitrary spline degree, taking advantage of its characteristics of excellent interpolation and approximation, multiscale representation, algorithm speed, and mesh-free construction. The problem of mathematical modeling in noninvasive medical imaging can be accurate and effective. Moreover, Chi et al. (2008) proposed a new CT image preprocessing method that uses a cubic spline curve to perform contour fitting on CT image data. This method has a good representation of detailed information. In addition, Albay and Kamaşak (2015) and Bahri et al. (2018) proposed a method based on Fourier and gradient histograms to realize human detection. This method can construct delicate human image contours in surveillance images.

Furthermore, (Albay and Kamaşak, 2015) applied Fourier descriptors to classify the extracted dermoscopic image lesion boundaries, which improved the diagnosis rate of the disease. Equally important, Serpa-Andrade et al. (2015) proposed an automated diagnosis system that uses the fast Fourier transform of shape features and the Hu moment descriptor to construct an esophageal image and uses the translation, rotation, and zoom invariance of the descriptor to transform the esophagus image. By classifying and using the irregularity of the z-line to describe the disease image, the method distinguishes a healthy esophagus from an esophagus with esophagitis. The literature review shows that the spline curve method and the Fourier method are two commonly used methods for modeling the contours of biological tissues.

In addition, Serpa-Andrade et al. (2015) proposed a local linear fitting function method combined with a DnCNN, which can improve the image quality and improve the results of medical image reconstruction. Although this method can suppress the error caused by noise, it is not suitable for the nerve bundle study conducted in this paper—research on the regularity of contour modeling and model parameters. Furthermore, Li (2019)proposed a multiple ellipse fitting framework suitable for densely connected contours. The framework decomposes complex multiellipse fitting tasks into single ellipse fitting, anomaly detection, and other easy-to-implement subtasks. The sliding window method and anomaly detection technology can extract multiple ellipses from the contour with good accuracy and efficiency. However, this method has two limitations: because the size of the sliding window is fixed, this method is not applicable when the size of the ellipse in the contour changes significantly; the frame cannot distinguish between an inner ellipse and an outer ellipse. Kapoor et al. (2019) used a polynomial curve to fit the contour of the iris, effectively realizing the positioning of the iris at any distance and direction. This method can be further extended to a mature iris recognition system to improve the efficiency of iris recognition, but there is no further explanation of the modeling effect of the iris contour.

On the basis of completing the acquisition and three-dimensional reconstruction of the nerve bundle contour in the peripheral nerve MicroCT image, in this paper, we constructed the mathematical model of the neural bundle contour in the framework of the Fourier transform and studied the statistical law of the parameters in the model. First, this paper constructed the neural bundle contour discrete point dataset; second, the quasi-uniform 3rd-order B-spline curve method and the Fourier transform method were used to construct the neural bundle contour model; third, the Dice coefficient was used as the evaluation index to explore the appropriate order in the established model based on the Fourier transform idea. The mathematical model built based on the appropriate order should make the Dice coefficient of all the modeled contours reach more than 95%. Fourth, the concept of the relative error of the Hausdorff distance was proposed and used to evaluate the contour modeling error of the nerve bundle.

## Methods and Results

### Study Design

The studies involving human specimens were reviewed and approved by the institutional review board of the Shenzhen Sixth People's Hospital.

**Figure 1:**
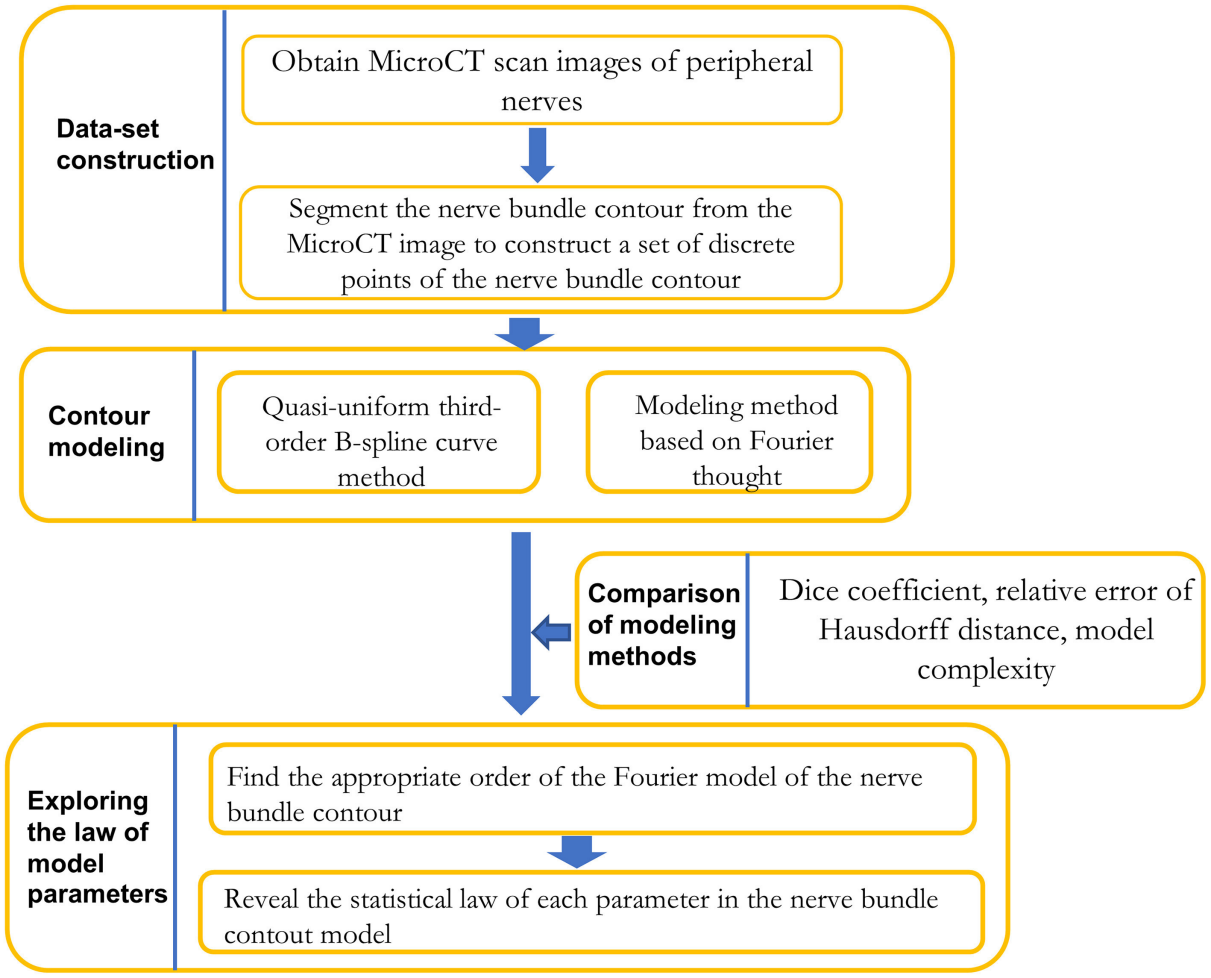
Framework for contour modeling method research on nerve bundles.

As shown in Figure 1, the research framework of this paper is divided into three stages: dataset preparation, model construction method research, and model parameter regularity exploration.

In the dataset preparation stage, a dataset of discrete points of nerve bundle contours was constructed based on segmenting nerve bundle contours.

In the research phase of the model construction method, the quasi-uniform 3rd-order B-spline curve method and the Fourier method were used to construct the nerve bundle contour. The two methods in terms of modeling method error, error, and adaptability to follow-up research were compared, and a more suitable modeling method was acquired.

In the exploration stage of the regularity of model parameters, according to the requirements of meeting the error requirements and the minimum model complexity, the lowest order of the model was obtained through experiments, and the probability density function of each model parameter was constructed by performing probability statistical analysis. The statistical law of each parameter was acquired.

### Dataset Construction

To explore the modeling method suitable for the nerve bundle profile and the law of model parameters, the following material preparations were carried out.

**Figure 2:**
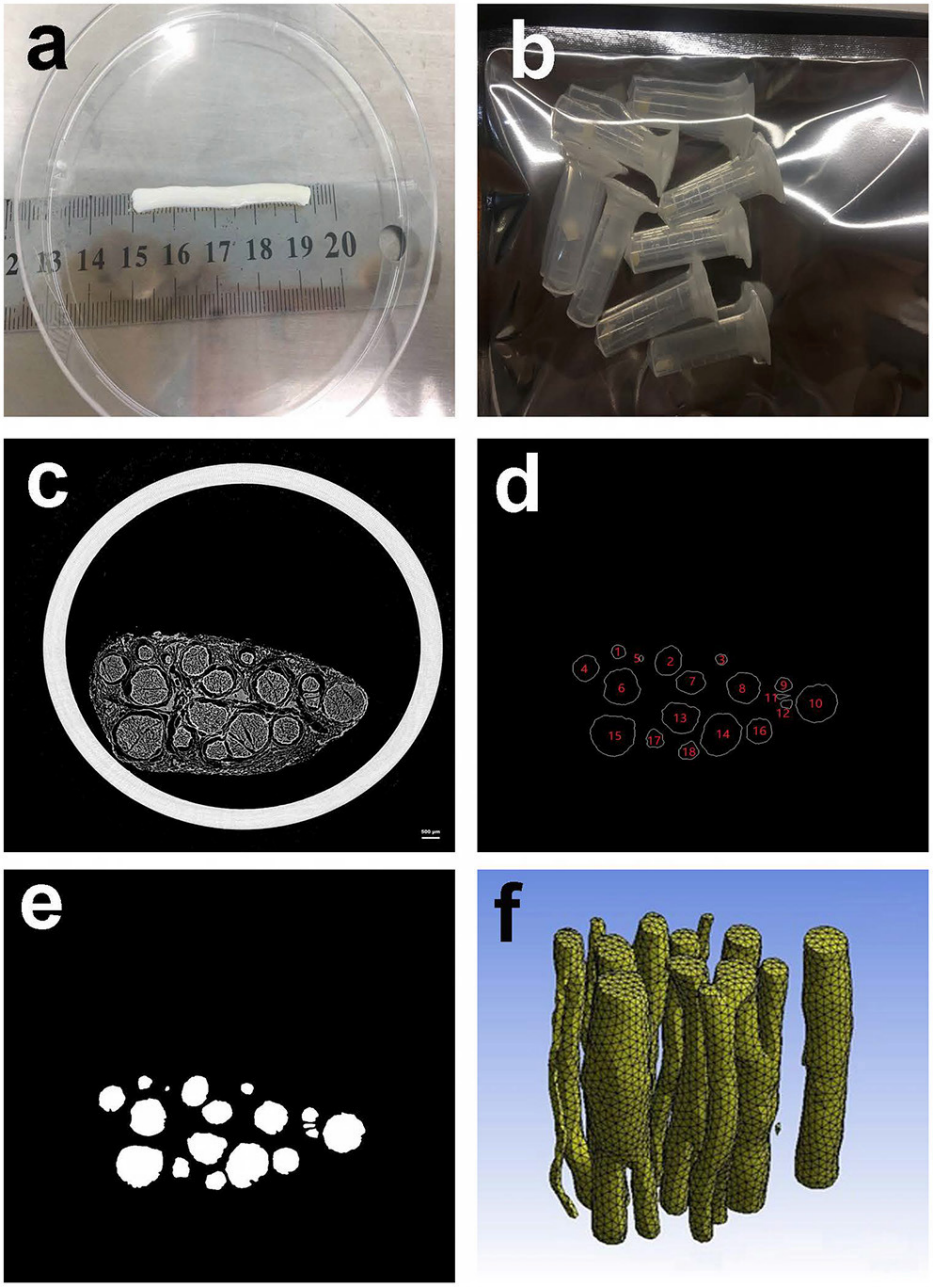
Preparing materials. **(a)** Peripheral nerve; **(b)** Peripheral nerve specimens with a length of ~3 mm; **(c)** 10th MicroCT image of the second specimen; **(d)** Contours of the nerve bundle from 10th MicroCT image; **(e)** Binary image of the nerve bundles from the 10th MicroCT image; **(f)** 3D reconstruction result of the second specimen.

Acquisition of specimens. The obtained peripheral nerves of the extremities are shown in Figure 2a. The peripheral nerves were dehydrated and frozen and then cut into multiple small sections with a length of ~3 mm as specimens, as shown in Figure 2b.

The specimen shown in Figure 2b was freeze-dried at −80° for 48 h, and the second specimen was arbitrarily selected as a case.

The second specimen was scanned by the MicroCT device, and a total of 522 MicroCT scan images were obtained. The 10th scan result image selected arbitrarily is shown in Figure 2c. The scanning parameters of MicroCT can be found in the literature (Zhu et al., 2016).

Image processing and deep learning methods were utilized to obtain nerve bundle contours (Zhong et al., 2015). A total of 10,962 nerve bundle contours were obtained from the sequence of scanned images. Taking the 10th scan result image as an example, the contour acquisition results are shown in Figure 2d. For clarity of subsequent expression, the nerve bundles in Figure 2d are numbered. The result of the binarization of the nerve bundle region is shown in Figure 2e.

To avoid the influence of the position coordinates on the neural bundle contour modeling, the centroid of each nerve bundle contour image was used as the coordinate origin for unified processing. All the discrete point sets of nerve bundle contours were saved one by one until the nerve bundle contour coordinate data of all images were obtained, and the nerve bundle contour coordinate discrete point dataset required in this article was constructed.

According to the image binarization processing result of the nerve bundle region, the nerve bundle was reconstructed in three dimensions, and the result is shown in Figure 2f. Figure 2f shows that the spatial structure of the nerve bundle is highly complicated, and the nerve bundle will split/merge several times within a distance of 1–5 mm. For this reason, this article only focused on the modeling and model parameter law study of the nerve bundles in the non-splitting/merging phase.

### Contour Modeling

#### Modeling Method of Nerve Tract Contour

Figures 2c–e shows that the contours of the nerve bundles in the peripheral nerves at the non-splitting/merging phase are circular-like figures with different areas, and the boundary contours are closed curves. According to the literature, the contour modeling of biological tissues usually adopts the B-spline curve method.

#### B-Spline Curve Modeling Method

The B-spline curve method is often used to construct curve models in computer-aided geometric design, which can effectively meet the requirements of graphic shape representation and geometric design, is convenient for shape information transmission and image shape mathematical modeling, and can solve the modeling problem of free-form curves. To construct a B-spline curve model, a starting point, an ending point, and multiple control points must be defined. By changing the coordinates of the vertices of the polygon inscribed in the contour (i.e., control points), the shape of the B-spline curve can be changed accordingly to realize nerve bundle contour modeling (Zhong et al., 2021).

The k-th B-spline model is:


(1) $$\begin{array}{r} c\left(u\right)=\overset{n}{\underset{i=0}{\sum }}{\text{P}}_{i}\cdot N_{i,k}\left(u\right)\;,u\in \left[0,1\right] \end{array}$$

where Pi(i = 0,1,2...n) represents the control point coordinates, and Ni, k(u)(i = 0,1,2...n) is the K-order canonical B-spline basis function. The basis function is a piecewise polynomial of K-order. The Cox-de Boor recurrence formula is usually used to express the B-spline basis function, which is defined as,


(2) $$\{\begin{array}{c} N_{i,0}(u)=\{\begin{array}{cc} 1, & u_{i}\leq u<u_{i+1}\\ 0, & other \end{array}\;\\ N_{i,k}=\frac{u-u_{i}}{u_{i+k}-u_{i}}N_{i,k-1}(u)+\frac{u_{i+k+1}-u}{u_{i+k+1}-u_{i+1}}N_{i+1,k-1}(u) \end{array}$$

Definition: In the formula of , the first subscript i of the N_*i, k*_ double subscript is the node number, the second subscript *k* is the number of the basis function, and the *k*-th B-spline curve is C^k−r^ continuous at the node where the repeatability is r.

In Formula 2, the meaning of u_*i*_ is as follows: suppose U is a set of n + k + 1 nondecreasing numbers (***U***: *u*_0_ ≤ *u*_1_ ≤ ... ≤ u_*n*+*k*+1_), ui is called a node, and set U is called a node vector. The interval [*u*_i_,*u*_i+1_] is the i-th node interval. If a node appears r times, this node represents multiple nodes with a repeat degree of r, written as *u*_i_(*r*).

To obtain the result of the quantitative description of the closed B-spline curve, two control points, **P**_n+1_=**P**_1_ and **P**_n+2_=P_2_, need to be added at the end of each contour dataset to make the curve connect end to end.

Based on the B-spline model, this paper used the quasi-uniform cubic B-spline curve method to describe the contour of the nerve bundle. The reasons are as follows:

1. Selection of the order: take *k* = 3, and the nodes that satisfy the repetition degree of 1 (*r* = 1) are **C**^2^ continuous; that is, the cubic B-spline curve is used.
2. Selection of the node vector: The node vector ***U*** must take a strictly increasing sequence to satisfy the repetition degree at the node of 1 (*r* = 1). A uniform B-spline model can be used when the node vector is uniformly distributed along the parameter axis.
3. Since the number of pixels forming the contour of the nerve bundle is not equal, to reduce the number of parameters/items of the model and obtain a description model with the same length of the number of parameters/items, a method of limiting the number of control points is adopted; that is, the dataset is sampled at equal intervals for a limited number of times. This method is a quasi-uniform sampling dataset method. In this paper, the quasi-uniform 3rd-order B-spline method is used to establish the model of the nerve bundle contour.

#### Modeling Method Based on the Fourier Transform

According to the idea of the Fourier transform, any periodic function can be expanded into the Fourier series of trigonometric functions, and each coefficient in the series determines the shape of the periodic function curve. Analyzing the contour of the nerve bundle shows that if the contour of the nerve bundle is converted to the complex plane, the contour is periodic in the complex plane, and the Fourier method can be used to construct the contour model. In this way, the follow-up exploration of the regularity of the nerve bundle profile is transformed into the exploration of the regularity of the Fourier model parameters.

**Figure 3:**
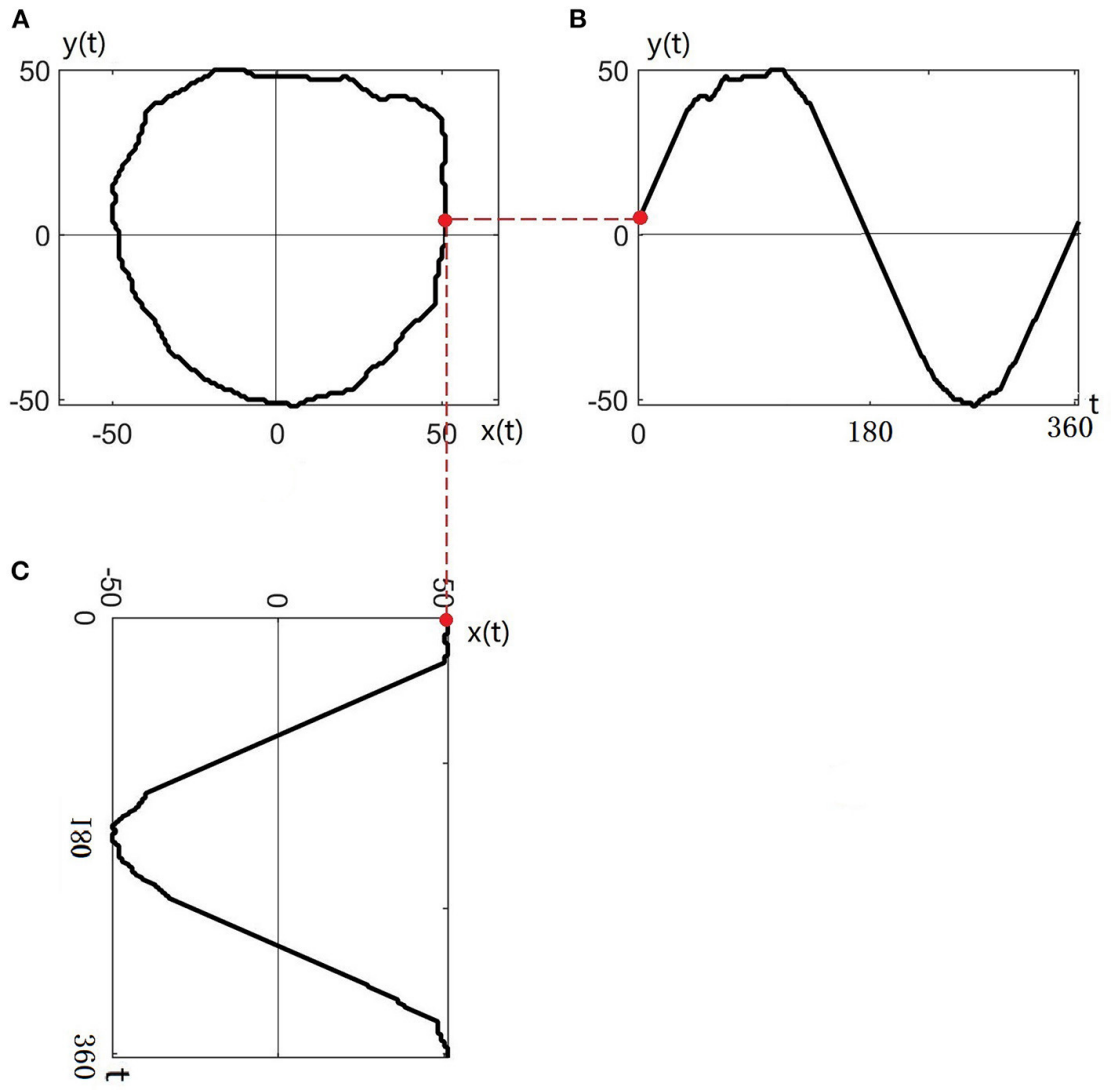
Transferring the contour of the nerve bundle to the complex plane: **(A)** Original contour of the nerve bundle; **(B)** Contour profiled to the *y*(*t*) plane; **(C)** Contour profiled to the *x*(*t*) plane.

The contour of any nerve bundle is transformed to the complex plane and named *z*(*t*), as shown in Figure 3A. Taking the centroid of the nerve bundle as the origin of the coordinate system, the nerve bundle contour *z*(*t*) can be expressed in the complex plane as:


(3) $$\begin{array}{r} z\left(t\right)=x\left(t\right)+iy\left(t\right) \end{array}$$

Where *x*(*t*), *y*(*t*) are two parameter planes orthogonal to each other.

Taking the intersection of the nerve bundle contour and the real axis as the starting point, the nerve bundle contour is circled 360° in a counterclockwise direction to obtain the projection curve of the nerve bundle contour on the real plane *x*(*t*) and imaginary plane *y*(*t*), as shown in Figures 3B,C.

Since the nerve bundle contour curve *z*(*t*) is a closed curve, the real and imaginary parts (*x*(*t*) and *y*(*t*), respectively) are periodic functions. Any periodic function can be represented by an infinite series composed of a sine function and a cosine function, so *z*(*t*) is expanded in a triangular Fourier series within 1 cycle (360°):


(4) $$x(t)y(t)=\left[\begin{array}{c} a_{0}\\ c_{0} \end{array}\right]+\sum_{k=1}^{\infty }\left[\begin{array}{cc} a_{k} & b_{k}\\ c_{k} & d_{k} \end{array}\right]\left[\begin{array}{c} \cos\frac{2k\pi t}{T}\\ \sin\frac{2k\pi t}{T} \end{array}\right]$$

(5) $$\left\{ \begin{array}{l} a_{k}=\frac{T}{2k^{2}\pi^{2}}\sum_{p=1}^{n}\frac{\Delta x_{p}}{\Delta t_{p}}(\cos\frac{2k\pi t_{p}}{T}-\cos\frac{2k\pi t_{p-1}}{T});\\ b_{k}=\frac{T}{2k^{2}\pi^{2}}\sum_{p=1}^{n}\frac{\Delta x_{p}}{\Delta t_{p}}(\sin\frac{2k\pi t_{p}}{T}-\sin\frac{2k\pi t_{p-1}}{T});\\ c_{k}=\frac{T}{2k^{2}\pi^{2}}\sum_{p=1}^{n}\frac{\Delta y_{p}}{\Delta t_{p}}(\cos\frac{2k\pi t_{p}}{T}-\cos\frac{2k\pi t_{p-1}}{T});\\ d_{k}=\frac{T}{2k^{2}\pi^{2}}\sum_{p=1}^{n}\frac{\Delta y_{p}}{\Delta t_{p}}(\sin\frac{2k\pi t_{p}}{T}-\sin\frac{2k\pi t_{p-1}}{T}). \end{array}\right.$$

Therefore:


(6) $$\left\{ \begin{array}{l} \;a_{0}=\frac{1}{T}\sum_{p=1}^{n}\left[\frac{\Delta x_{p}}{2\Delta t_{p}}(t_{p}^{2}-t_{p-1}^{2})+\xi_{p}(t_{p}-t_{p-1})\right];\\ c_{0}=\frac{1}{T}\sum_{p=1}^{n}\left[\sum \frac{\Delta y_{p}}{2\Delta t_{p}}(t_{p}^{2}-t_{p-1}^{2})+\delta_{p}(t_{p}-t_{p-1})\right]. \end{array}\right.$$

(7) $$\left\{ \begin{array}{l} \Delta t_{p}=\sqrt{\Delta x_{p}^{2}+\Delta y_{p}^{2}};t_{p}=\sum_{i=1}^{p}\Delta t_{i};\xi_{1}=\delta_{1}=0;\\ \xi_{p}=\sum_{j=1}^{p-1}\Delta x_{j}-\frac{\Delta x_{p}}{\Delta t_{p}}\sum_{j=1}^{p-1}\Delta t_{j};\delta_{p}=\sum_{j=1}^{p-1}\Delta y_{j}\\ \;-\frac{\Delta y_{p}}{\Delta t_{p}}\sum_{j=1}^{p-1}\Delta t_{j}. \end{array}\right.$$

where Δ*x*_*p*_ is the change in the *x*-direction, Δ*y*_*p*_ is the change in the *y*-direction, *p* is the current coordinate, and *k* is the order. In Formula 4, the model parameters *a*_0_, *c*_0_ represent the centroid coordinates of the nerve bundle contour; the model parameters *a*_*k*_, *b*_*k*_, *c*_*k*_, *d*_*k*_ determine the shape, rotation angle, and scale of the nerve bundle contour. The parameter *k* = 1 is the fundamental frequency component, and *k*≥2 is the harmonic component. In the nerve bundle contour model based on the Fourier transform idea (the following is referred to as the Fourier model of the contour), the low-frequency component describes the main shape of the nerve bundle contour, and the high-frequency component describes the details of the nerve bundle contour. Under the premise of satisfying the description accuracy, a small number of parameters can be used to describe the nerve bundle contour.

#### Methods to Mine the Regularity of Model Parameters

Observing the contour shape of the nerve bundle in Figure 2d, it can be seen that the contours of the nerve bundles in the same tomographic image are different, and the contours of the same nerve bundle on different slices are also different. After turning the regularity research problem of nerve bundle contours into the regularity research problem of model parameters, it is necessary to determine the proper order of the model through experimental methods. The condition for the proper order of the model requires that the order should be as low as possible under the premise of meeting the accuracy; that is, the model parameters should be as few as possible.

In addition, the statistical analysis methods of each parameter in the model include evaluating the normal distribution, logistic distribution, and *t* distribution with scale/location. Based on constructing the probability density function of each parameter, the following will explore the law of model parameters extending in space to achieve the goal of describing the spatial change law of the nerve bundle contour with a small number of parameters.

### Comparison of Modeling Methods and Exploring the Law of Model Parameters

#### Experimental Environment Configuration

In this paper, the software platform for constructing the neural tract contour model is JetBrains PyCharm Community Edition 2018.2.4 × 64 running on a Win10 64-bit operating system.

#### Evaluation Index

##### Overall Evaluation Index of the Contour Model: *Dice* Coefficient

The *Dice* coefficient is an evaluation index commonly used to compare the similarity of two samples (Roth et al., 2018), which is defined as:


(8) $$\begin{array}{r} Dice=\frac{2|S_{A}\cap S_{B}|}{\left|S_{A}|+|S_{B}\right|}\times 100\% \end{array}$$

In this paper, represent the original contour of the nerve bundle and the area enclosed by the contour curve constructed by the mathematical model, respectively. The larger the Dice coefficient is, the more similar the two contours are on a macroscopic scale. The *Dice* coefficient is used to evaluate the approximation degree of the nerve bundle contour constructed by the quasi-uniform 3rd-order B-spline curve method and the Fourier method compared with the original contour from the overall perspective. In this article, it is required that the *Dice* coefficient of the modeled contour and the actual contour be no <95%.

Because the *Dice* coefficient lacks the evaluation of the contour boundary details when evaluating the modeling accuracy, we also introduce the relative error *H*_*E* of the Hausdorff distance as the evaluation criterion of the contour detail error.

##### Local Error Evaluation Index of the Contour Model: Relative Error *H*_*E* of the Hausdorff Distance

The Hausdorff distance *H* is a measure to describe the similarity between two sets of points (Kim et al., 2016) and is defined as:

There are two sets of discrete data points A = {*a*_1_,*a*_2_*a*_2_,…,*a*_m_} and B = {*b*_1_,*b*_2_*b*_2_,…,*b*_n_}. In this paper, the discrete data point set A is the original contour discrete data point set, and point set B is a set of discrete data points of the reconstructed contour of the model. The Euclidean distance between point *b*_x_(x ∈ 1,2,…,n) in dataset B and all the corresponding points in dataset A was found, and then the minimum value was taken. The shortest vertical distance between point *b*_x_ and the original contour was obtained, that is, the boundary error *e*:


(9) $$\begin{array}{r} e_{x}=min_{y\in 1,2,\ldots ,m}\left(\left||b_{x}-a_{y}|\right|\right) \end{array}$$

The Hausdorff distance H is:


(10) $$\begin{array}{r} H=\underset{x\in 1,2,...,n}{\max}\left(e_{x}\right) \end{array}$$

Although the Hausdorff distance *H* can better evaluate the difference in contour details, the circumference of the nerve bundle contour is different. The same Hausdorff distance *H* has very different descriptions of nerve bundle contours with different circumferences. For this reason, this paper proposes using the relative error of the Hausdorff distance as the evaluation index.

Let the number of discrete points contained in a particular nerve bundle contour be N; then, the relative error of the Hausdorff distance *H*_*E* is defined as:


(11) $$\begin{array}{r} H\text{\_}E=\frac{H}{N}\times 100\% \end{array}$$

In this article, the relative error *H*_*E* of the Hausdorff distance is required to be no more than 5%.

#### Experimental Plan Design

##### Experiment 1

The methods suitable for constructing a neural tract contour mathematical model were explored: the quasi-uniform 3rd-order B-spline curve modeling method and Fourier modeling method were used to construct the mathematical model of the nerve tract contour, and the advantages and disadvantages were compared and analyzed.

##### Experiment 2

The proper order of the Fourier model was investigated. The Fourier models of different orders were taken to model the nerve bundle contour, the evaluation index was used to evaluate it, and the lowest order that meets and was selected as the appropriate order of the Fourier model, which not only ensures the accuracy of the model but also makes the model parameters as few as possible.

##### Experiment 3

The statistical law of profile model parameters of the nerve bundle was revealed: the Fourier model parameters *a*_0_, *a*_1_,…, *a*_4_, *b*_1_,…, *b*_4_, *c*_0_, *c*_1_,…, *c*_4_, *d*_1_,…, *d*_4_ and those of other models. Statistical analysis of parameters was performed, the probability density distribution of each parameter was fitted, and the probability distribution and probability density function were obtained.

#### Experimental Results

##### Experiment 1: A Mathematical Model Suitable for Constructing Nerve Bundle Contours Was Explored

**Figure 4:**
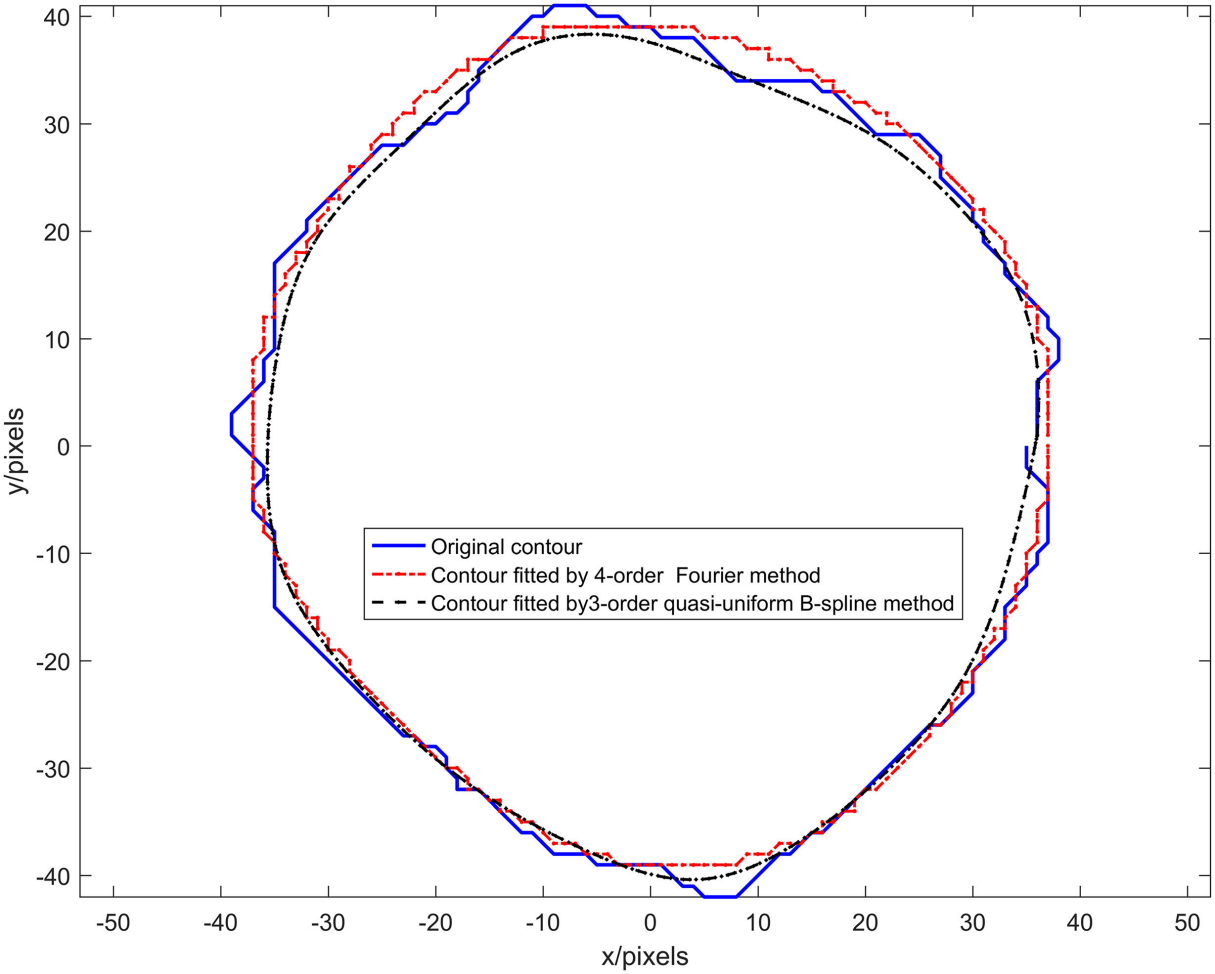
Results of experiment 1.

The quasi-uniform third-order B-spline curve method and the Fourier method were used to construct the neural bundle contour model, and the neural bundle numbered 8 in Figure 2d was used as a case to show the contour modeling results, as shown in Figure 4. It can be seen from Figure 4 that:

The nerve bundle contour constructed by the quasi-uniform 3rd-order B-spline method with 21 control points and the nerve bundle contour constructed by the 4th-order Fourier method are very close to the original contour.

When the number of control points is 21, the quasi-uniform third-order B-spline curve method is used to construct the neural bundle contour with a *Dice* coefficient of 96.93%, and the Hausdorff distance is <5 pixels *H*_*E* ≤ 0.5%.

The coefficient of the neural bundle profile constructed by the fourth-order Fourier method is 95.17%, and the Hausdorff distance is <4.27 pixels *H*_*E* ≤ 0.77%.

The adjustable parameters modeled by the fourth-order Fourier method are 16 (excluding the constant term for locating the centroid coordinates of the nerve bundle), and the adjustable parameters modeled by the third-order quasi-uniform B-spline curve method are more than 64.

The control point coordinates modeled by the quasi-uniform 3rd-order B-spline curve method depend on the discrete point set of the nerve bundle contour. In particular, the perimeter of the nerve bundle contour is different since the data in the discrete point concentration are very different. Therefore, the data obtained by uniformly sampling 21 times in this discrete point concentration have no physical meaning. The model parameters modeled by the Fourier method represent the rotation process of a contour point in the complex plane *z*(*t*) around the center of mass. The angle and period corresponding to the *x*(*t*)-plane and the *y*(*t*)-plane are not related to the contour circumference of the nerve bundle and have a clear physical significance. Moreover, using the Fourier method to construct a nerve bundle contour model can obtain a model with the same number of parameters when the nerve bundle contour circumference is different, which is extremely important for follow-up research.

Comprehensively comparing the above factors, we choose the Fourier method as the primary modeling method of nerve bundle contours.

##### Experiment 2: Finding a Suitable Fourier Model Order

The Fourier method with different orders has different accuracies in describing the contours of nerve bundles. The higher the order is, the higher the accuracy, the more the model parameters, and the higher the model complexity. The complexity of the model is too high, which is not conducive to the subsequent exploration of the regularity of model parameters. In this article, the specimen shown in Figure 2c is used as a case to illustrate the experimental process as follows.

**Figure 5:**
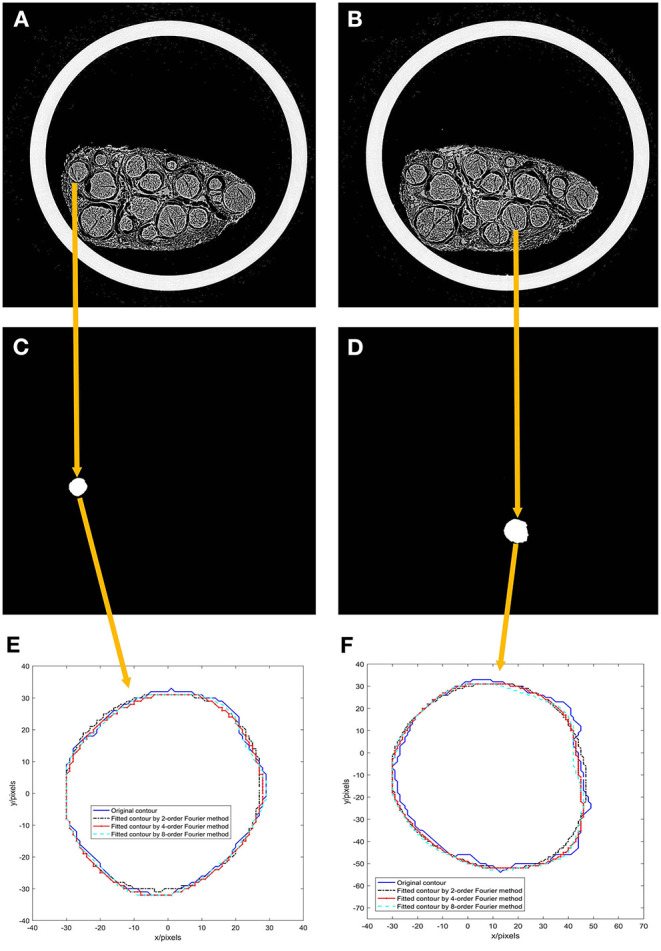
Results of experiment 2. **(A)** 1st image of the 522 scanned sequence images of the specimen; **(B)** 46th image of the 522 scanned sequence images of the specimen; **(C)** A binarized image of the 1st scanned image; **(D)** a binarized image of the 46th scanned image; **(E)** Fourier models of the 1st scanned image; **(F)** Fourier models of the 46th scanned image.

The first and 46th images among the 522 scanned sequence images of the specimen were chosen, as shown in Figures 5A,B.

A nerve bundle is chosen in Figures 5A,B, the algorithm is used to extract its contour, and a binarized image is obtained, as shown in Figures 5C,D.

The contours of the two nerve bundles were modeled using the 2nd-, 4th-, and 8th-order Fourier methods, and the results are shown in Figures 5E,F.

From the modeling results in Figures 5E,F, it can be seen that the curve fitted by the second-order Fourier model has a relatively significant difference from the original contour. Moreover, the curve fitted by the fourth-order Fourier model has a relatively small difference from the original contour. The curve fitted by the eighth-order Fourier model is very close to the original contour.

The second-, fourth-, and eighth-order Fourier methods were used to model all nerve bundle profiles, and their average *Dice* coefficients, Hausdorff distance H, and Hausdorff distance relative error were calculated. The results are shown in Table 1.

**Table 1:** Calculation results of evaluation indices by modeling different orders of the Fourier method.

**Order (*k*)/**	**1**	**2**	**3**	**4**	**5**	**6**	**7**
*Dice* (mean value)/%	93.29	94.20	94.73	95.17	95.55	95.95	96.22
*Hausdorff distance H/pixel*	11.70	7.81	7.07	5.83	5.83	5.10	5.38
Relative error *H*_*E*/%	1.07	0.93	0.84	0.77	0.71	0.64	0.61

Analysis of the data in Table 1 shows the following:

1. When the order of the Fourier method reaches or exceeds order 4, the *Dice* coefficient of the model is higher than 95%. Taking the 95% confidence level as the criterion, a Fourier model of order 4 or higher should be selected for neural bundle contour modeling.
2. The relative error of the Hausdorff distance modeled by Fourier methods above the fourth order is <1%. In detail, the contours modeled by the Fourier method of each order are close to the actual contours.
3. Comparing the data of the fourth-order model and the fifth-order model in Table 1, it is found that the Hausdorff distance of the two models is the same. Comparing the data of the 6th order and 7th order models in Table 1, it is found that the Hausdorff distance of the 6th order model is lower than the Hausdorff distance of the 7th order model. It can be seen that the lower the order of the model is, the more minor the modeling error; that is, the order of the mathematical model and the Hausdorff distance is not monotonic.

In summary, we choose the 4th order as the appropriate Fourier model order, and the modeling contour is stable with *Dice*≥95% and *H*_*E* ≤ 5%. This ensures the model's accuracy and makes the model parameters as few as possible and the model complexity as low as possible.

##### Experiment 3: Statistical Analysis of Model Parameters

The 4th-order Fourier model was used to model the contours of 10,962 nerve bundles in 552 scanned images, and data of 18 model parameters, such as *a*_0_, *a*_1_,…, *a*_4_, *b*_1_,…, *b*_4_, *c*_0_, *c*_1_,…, *c*_4_, *d*_1_,…, *d*_4_, etc., were obtained. Statistical analysis of the data for each parameter was performed, and the results are as follows.

The constant term parameters *a*_0_ and *c*_0_ are used to locate the center of mass coordinates of the nerve bundle contour area and have no effect on the contour shape of the nerve bundle.

**Figure 6:**
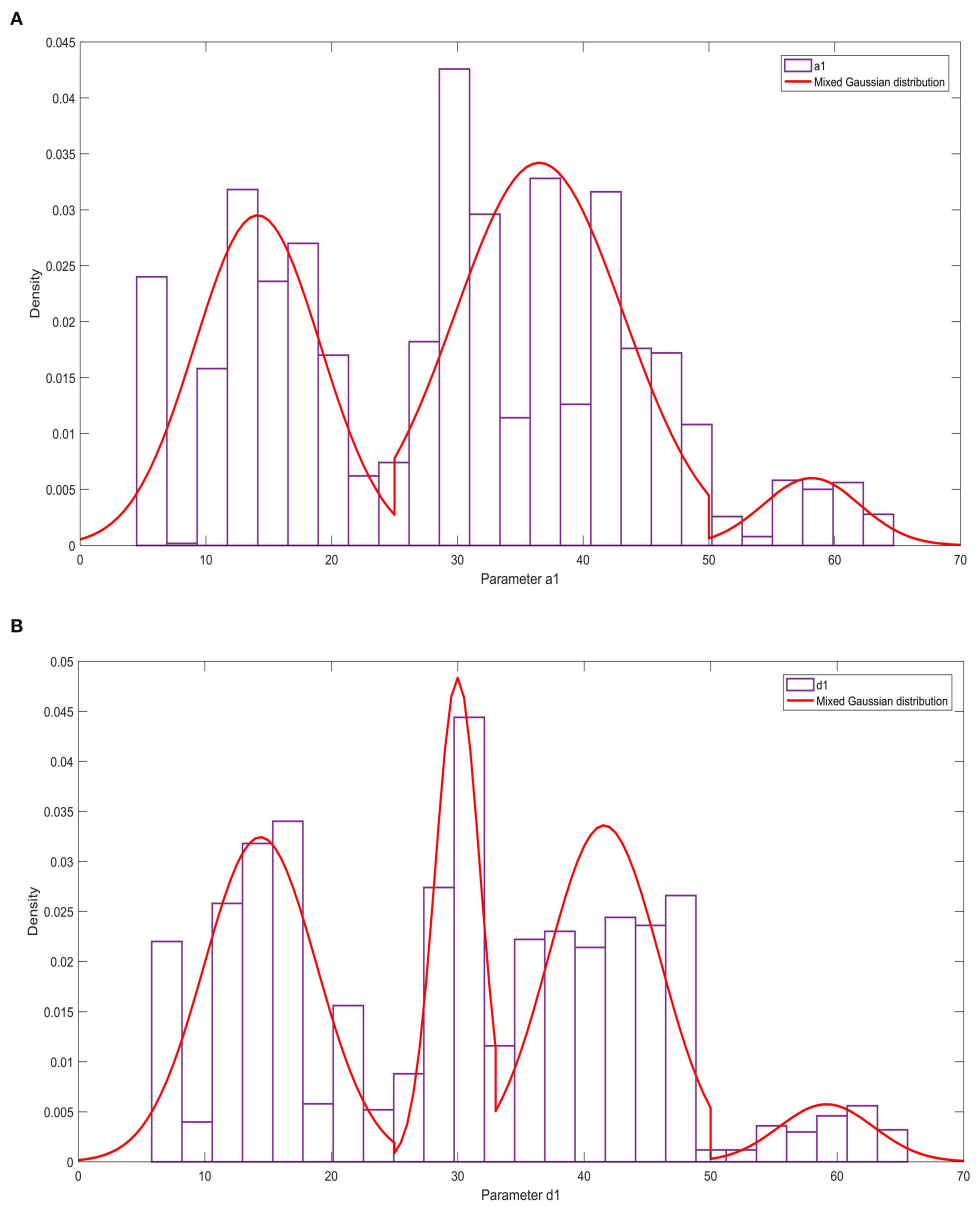
The statistical law of parameters a1 and d1. **(A)** Histogram and probability density function of parameter a1; **(B)** Histogram and probability density function of parameter d1.

The relevant results of the histogram of the parameters *a*_1_ and *d*_1_ and the probability density function are shown in Figure 6. Figure 6 shows that parameter *a*_1_ has three peaks, and parameter *d*_1_ has four peaks. These two parameters obey the mixed Gaussian distribution. To this end, the Gaussian mixture model is used to construct the probability density functions of the parameters *a*_1_ and *d*_1_,


(12) $$\{\begin{array}{c} \begin{array}{cc} \frac{1}{5\sqrt{2\pi }}e^{\frac{{\left(x-14.11\right)}^{2}}{50}} & \left(0\leq x<25\right) \end{array}\\ \begin{array}{cc} \frac{1}{6.68\sqrt{2\pi }}e^{\frac{{\left(x-36.51\right)}^{2}}{89.2448}} & \left(25\leq x<50\right) \end{array}\\ \begin{array}{cc} \frac{1}{3.86\sqrt{2\pi }}e^{\frac{{\left(x-58.15\right)}^{2}}{29.7992}} & \left(50\leq x\right) \end{array} \end{array}$$

as shown in Formulas 12, 13:


(13) $$f_{d_{1}}(x)=\{\begin{array}{c} \begin{array}{cc} \frac{1}{4.44\sqrt{2\pi }}e^{\frac{{\left(x-14.4\right)}^{2}}{39.4272}} & \left(0\leq x<25\right) \end{array}\\ \begin{array}{cc} \frac{1}{1.77\sqrt{2\pi }}e^{\frac{{\left(x-29.99\right)}^{2}}{6.2658}} & \left(25\leq x<33\right) \end{array}\\ \begin{array}{cc} \frac{1}{4.41\sqrt{2\pi }}e^{\frac{{\left(x-41.57\right)}^{2}}{38.8962}} & \left(33\leq x<50\right) \end{array}\\ \begin{array}{cc} \frac{1}{3.75\sqrt{2\pi }}e^{\frac{{\left(x-59.15\right)}^{2}}{28.125}} & \left(50\leq x\right) \end{array} \end{array}$$

**Figure 7:**
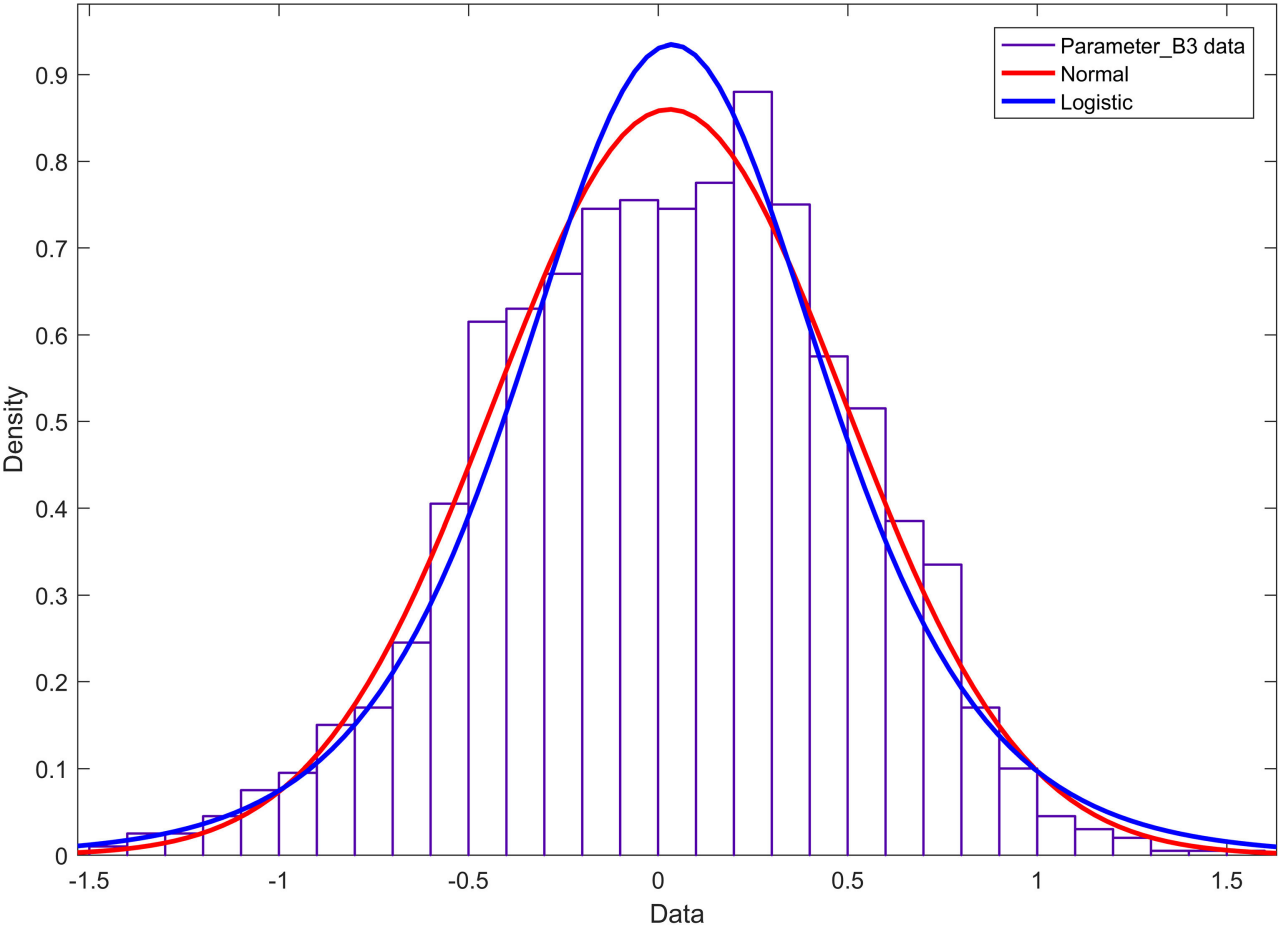
Histogram and probability density function of parameter b3.

The histogram and cumulative probability density function results of parameter *b*_3_ are shown in Figure 7. Figure 7 shows that the normal and logistic distributions fit the histogram of parameter *c*_1_. The results show that parameter *b*_3_ obeys the normal distribution of *N*(0.03, 0.46^2^).

**Figure 8:**
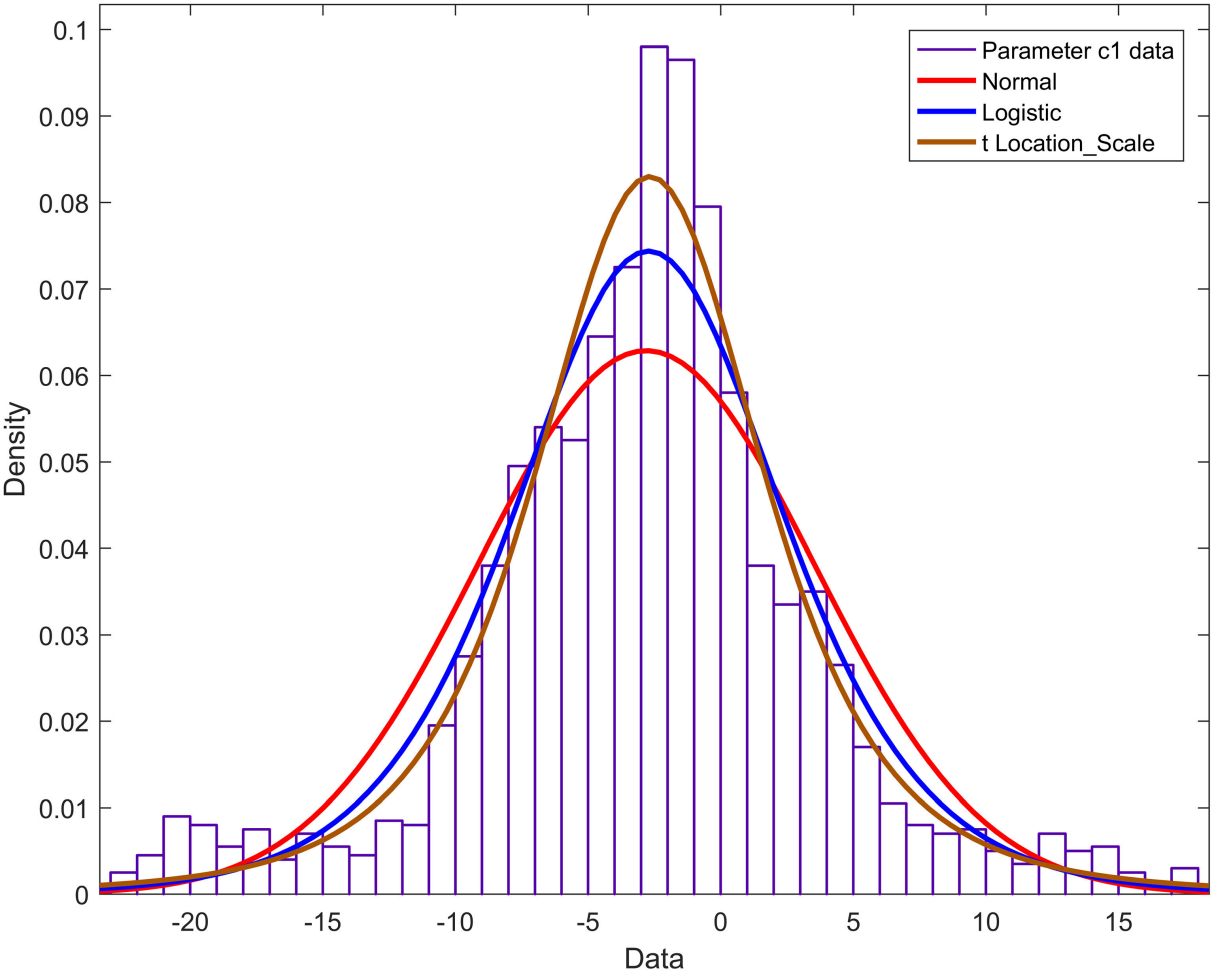
Histogram and probability density function of parameter c1.

The probability density functions of the remaining 13 Fourier model parameters a2,…, a4,…, b1,…, b2,…, b4,…, c1,…, c4,…, d2,…, d4 are constructed. The parameter *c*_1_ is taken as a case description. The normal, logistic, and t distributions with scale/location were used to fit the histogram of parameter *c*_1_, and the results are shown in Figure 8. Figure 8 shows that the cumulative probability density function curve with the scale/location parameter t distribution is closest to the actual probability density curve. Therefore, parameter *c*_1_ obeys the t distribution with scale/location parameters, and the probability density function is:


(14) $$\begin{array}{r} f_{T}\left(x\right)=\frac{\Gamma \left(\frac{\upsilon +1}{2}\right)}{\sqrt{\upsilon \pi }\cdot \Gamma \left(\frac{\upsilon }{2}\right)}{\left(1+\frac{t^{2}}{\upsilon }\right)}^{-\frac{\upsilon +1}{2}} \end{array}$$

Among them is the gamma function.

The position, scale, and degree of freedom distribution of the 13 parameters *a*_2_,…, *a*_4_, *b*_1_, *b*_2_, *b*_4_, *c*_1_,,…, *c*_4_, *d*_2_,… *d*_4_ of the neural bundle profile Fourier model are shown in Table 2.

**Table 2:** Calculation results of evaluation indices by modeling different orders of the Fourier method.

**Parameter**	***a*_2_**	***a*_3_**	***a*_4_**	***b*_1_**	***b*_2_**	***b*_4_**	***c*_1_**	***c*_2_**	***c*_3_**	***c*_4_**	***d*_2_**	***d*_3_**	***d*_4_**
Position μ	−0.08	−0.05	0.01	2.36	−0.03	0.02	−2.67	0.02	0.02	−0.01	0.04	0.18	0.11
Scale σ	0.66	0.49	0.36	4.45	0.49	0.40	4.48	0.47	0.47	0.30	0.62	0.40	0.36
Variance υ	5.43	5.7	7.17	2.85	2.62	4.84	3.45	2.37	16.71	3.18	4.44	5.39	4.33

In Table 2, the parameters *b*_1_ and *c*_1_ are the parameters of the fundamental component in the Fourier model, and the other parameters are the corresponding parameters of the harmonic components in the Fourier model. By observing the scale σ row of Table 2 and comparing the scale σ of the parameters *b*_1_ and *c*_1_ with the scale σ of other parameters, the findings indicate that the scale parameter σ value of the fundamental component is much larger than the scale parameter σ value of the harmonic component. This shows the probability of the parameters *b*_1_ and *c*_1_. The density function curve is relatively flat, and the probability density function curve of the harmonic component parameter value is sharp. The concentration of the harmonic component parameter value is much higher than the concentration of the parameters *b*_1_ and *c*_1_.

## Discussion

In repairing peripheral nerve injury, if the nerve bundle can be docked according to its original spatial structure, the disability rate will be significantly reduced. However, there are currently no technical means to achieve precise docking. Constructing a mathematical model of the inner nerve bundle contour of the peripheral nerve and studying the regularity of the model parameters is critical to achieve a precise connection of the peripheral nerve. To this end, Zhong et al. (2015) frozen the median nerve, followed by sectioning, microscopic imaging, registration, extraction of nerve bundle contours, and 3D reconstruction to obtain a visual model of the nerve bundle. Since this method requires slicing and registration, the efficiency and accuracy are not high. To this end, Zhu et al. (2016) used MicroCT to image peripheral nerves, and Zhong et al. (2021) used Mask RCNN method to extract nerve bundle contours from these images, and obtained long-segment inner nerve bundles Methods of 3D reconstruction. In order to explore the extension law of nerve bundles in space, Zhong et al. (2020) simplified the shape of nerve bundles in space to their centroid space curves, and used the Fourier method to build a mathematical model, and obtained the nerve bundles. The space shape law. In order to accurately connect the nerve bundles, it is necessary to know many key parameters of the nerve bundles, including: the morphological parameters of the nerve bundles and their spatial extension laws, and the mathematical model of the nerve bundle contours. To this end, Zhong et al. (2021) studied the statistical laws of core morphological parameters such as area, perimeter, and roundness of nerve bundles, as well as the laws of spatial extension. In this paper, by constructing a mathematical model of the nerve bundle contour and obtaining the statistical law of the model parameters, it lays a key foundation for the accurate docking of the nerve bundle.

In this paper, the quasi-uniform third-order B-spline curve method and the Fourier transform method are used to construct the neural bundle contour model, and the regularity of the model parameters is studied. Experiment 1, shown in Figure 5, demonstrates that the quasi-uniform 3rd-order B-spline curve method used to construct the neural bundle contour has a coefficient of 96.93%, and the Hausdorff distance is less than 5 pixels. The coefficient of the neural bundle profile constructed by the fourth-order Fourier method is 95.17%, and the Hausdorff distance is <4.27 pixels. It can be seen that (1) both the quasi-uniform 3rd-order B-spline curve method and the Fourier method can construct the nerve bundle contour model. (2) Although the modeling accuracy of the Fourier model is slightly lower than that of the quasi-uniform 3rd-order B-spline curve model, the complexity of the contour Fourier model is much lower than that of the quasi-uniform 3rd-order B-spline curve model. (3) The control point coordinates of the quasi-uniform 3rd-order B-spline curve model are obtained by uniformly sampling the original data and have no physical meaning; each parameter of the Fourier model expresses the detailed shape of the nerve bundle profile at different orders and has a clear physical meaning. Therefore, the Fourier method is more suitable for constructing nerve bundle contour models.

According to the results of Experiment 2, it can be seen that the Dice coefficient can reach more than 95% when the Fourier model of order four and above is used to construct the nerve bundle contour curve, and the relative error of the Hausdorff distance is <0.77%. There is no monotonic relationship between the Hausdorff distance and model order. Considering model complexity and modeling accuracy, it is appropriate to use the 4th-order Fourier method to construct the mathematical model of the nerve bundle contour.

According to the results of Experiment 3, the research in this paper shows that when the 4th-order Fourier model is used to model the nerve bundle profile, the two fundamental parameters a1 and d1 of the model obey the mixed Gaussian distribution, with a relatively obvious 3–4 a wave peak. The degree of freedom of the harmonic parameter b3 is much larger than that of other parameters, and it obeys the normal distribution. The peak of the probability density function is lower, and the two ends are slightly higher than other parameters. The other two fundamental wave parameters b1, c1, and other harmonic parameters in the model all obey the *t*-distribution with position/scale parameters, and the probability density function has a relatively sharp peak shape. On this basis, when we need 3D printing or 3D reconstruction of nerve bundles, the probability distribution corresponding to the parameters can be used to randomly generate an actual value. Substitute this value into the mathematical model constructed in this paper, and then we can obtain the nerve bundle with a difference of <5% from the original nerve bundle profile. It can be seen that the construction of the nerve bundle outline can provide a positioning reference for the docking nerve. It can provide a smooth walking trajectory for 3D printing, and can also significantly reduce the storage space required to save nerve bundle information, so as to express the contour information of long nerve bundles with a small number of model parameters and ensure accuracy. And lay the foundation for the follow-up exploration of the regularity of neural bundles extending in space.

## Conclusion

Constructing the contour of the nerve bundle can provide a positioning benchmark for peripheral nerve repair surgery. For this reason, based on constructing the nerve tract contour discrete point dataset, this paper explores a method suitable for constructing the neural tract contour mathematical model in the non-splitting and merging stage of peripheral nerve MicroCT images.

The main contributions of this paper are as follows: (1) Aiming at the modeling problem of nerve bundle contours in peripheral nerve MicroCT images, a modeling method based on the Fourier transform is proposed and compared with the classic B-spline curve modeling method. (2) Given the difficulty in evaluating the local description accuracy of the neural bundle contour mathematical model, the concept of the relative error of the Hausdorff distance is proposed. (3) Using the Dice coefficient as the evaluation index, we found the appropriate Fourier model order with the required accuracy and low complexity. (4) The statistical law of each parameter in the Fourier model of the nerve bundle profile was revealed.

Next, we will explore the correlation between model parameters and spatial extension.

## Data Availability Statement

The original contributions presented in the study are included in the article/supplementary materials, further inquiries can be directed to the corresponding author.

## Ethics Statement

The studies involving human specimen were reviewed and approved by the Institutional Review Board of the Shenzhen Sixth People's Hospital.

## Author Contributions

SZ: conceptualization and writing—original draft preparation. SZ, YZ, and ZT: methodology. SZ and YZ: validation and funding acquisition. YZ and SS: writing—review and editing. All authors have read and agreed to the published version of the manuscript.

## Funding

This research was funded by the National Natural Science Foundation of China (grant numbers 81801210, 61975248); Science and Technology Project of Guangzhou of China (grant numbers 202102020157, 202007040004); Natural Science Foundation Project of Guangdong Province, China (grant number 2018A0303130137); Major Science and Technology Project of Yunnan Province, China (grant number 202102AA100012).

## Conflict of Interest

The authors declare that the research was conducted in the absence of any commercial or financial relationships that could be construed as a potential conflict of interest.

## Publisher's Note

All claims expressed in this article are solely those of the authors and do not necessarily represent those of their affiliated organizations, or those of the publisher, the editors and the reviewers. Any product that may be evaluated in this article, or claim that may be made by its manufacturer, is not guaranteed or endorsed by the publisher.
